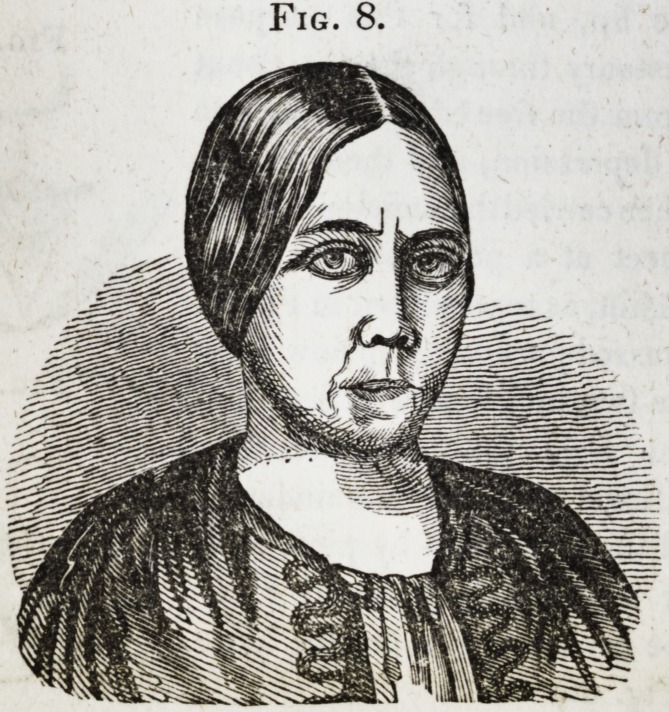# Case of Elongation of the under Jaw and Distortion of the Face and Neck, Caused by a Burn, Successfully Treated

**Published:** 1849-01

**Authors:** S. P. Hullihen

**Affiliations:** Wheeling, Va.


THE AMERICAN

### Journal of Dental Science.

Vol. IX.] JANUARY, 1849. [No. 2.

### ARTICLE I.

*Case of Elongation of the Under Jaw and Distortion of the Face and Neck, caused by a Burn, successfully treated.* Reported, with cuts, by Dr. S. P. Hullihen, of Wheeling, Va.

Miss Mary S——, aged 20, daughter of the Hon. Wm. S——, of Ohio, came to Wheeling in the spring of 1848, to obtain relief from the effects of a very severe burn, which she had received fifteen years before.

The burn was principally confined to the neck and lower part of the face, and its cicatrix produced a deformity of the most dreadful character. Her head was drawn forward and downward—the chin was confined within an inch of the sternum—the under lip was so pulled down that the mucous membrane of the left side came far below the chin—the under jaw was bowed slightly downward, and elongated, particularly its upper portion, which made it project about one inch and three-eighths beyond the upper jaw. In front there was scarcely any appearance of either chin or neck; she was unable to turn her head to either side; the cheeks and upper lip were dragged considerably downward; she could not close her eyelids; she could not close her jaws, but for an instant, and then only by bowing her head forward; she could not retain her saliva for a single instant, and, as might be expected, her articulation was very indistinct.

She had been taken to the city of New York, some years before, for the purpose of being relieved of this deformity, and was placed under the care of two of the most distinguished surgeons in that city, who performed an operation by dissecting up the cicatrix on the neck, then raising the head, and sliding up the cicatrix from its original position, leaving a raw surface below to heal up by granulation. I need scarcely add that the operation was entirely unsuccessful.

After a careful examination of the case, it became evident that such a complicated deformity could be best remedied by performing three separate operations: one upon the jaw; another upon the neck; and a third upon the under lip.

To remove the projection of the under jaw seemed to require the first attention. Unless that could be done, the other operations, however successful, would add but little if any to the personal appearance of the patient. This lengthening of the jaw had taken place entirely between the cuspidatus and first bicuspid tooth of the right side, and between the first and second bicuspides of the left. By this elongation, the teeth just described were separated on both sides about three-fourths of an inch. To saw out the upper edge of these elongated portions of the jaw, and then to divide that part of the jaw in front of the spaces thus made, by sawing it through in a horizontal manner, so as to permit the upper and detached portion to be set back in its proper and original position, appeared to be the only possible way of remedying the deformity. This plan I therefore adopted, and performed the operation on the 12th day of June, in the manner now to be described.

The operation was commenced by sawing out, in a V shape, the elongated portions, together with the first bicuspid on the left side, each section extending about three-fourths of the way through the jaw. I then introduced a bistoury at the lower point of the space from which the section was removed on the right side, and pushed it through the soft parts, close to and in front of the jaw, until it came out at the lower point of the space on the left side. The bistoury was then withdrawn, and a slender saw introduced in the same place, and the upper three-fourths of the jaw, containing the six front teeth, was sawed off on a horizontal line ending at the bottom of the spaces before named, (see Fig. 1,) the detached portions being

FIG. 1.

still connected, on the outer and inner sides, to the jaw below, by the soft parts. After having, with the bone-nippers, removed from the detached portion the corners which were created by the horizontal and perpendicular cuts of the saw, it was set back so that the edges, from which the V shaped sections were removed, came together, as represented in Fig. 2.

FIG. 2.

Thus it will be perceived that this portion of jaw and teeth, which before projected and inclined outward, now stood back and inclined inward, and in its proper and original place.

In this position the jaw was secured, by passing ligatures around the cuspidati in the detached portion, and the now adjoining bicuspides in the sound portion. Then taking an impression of the jaw in very soft wax, a cast was procured, and a silver plate struck up and fitted over the teeth and gum, in such a manner as to maintain the parts in that same relation, beyond the possibility of movement.

The patient declared that the operation gave her little or no pain. There was a little swelling about the chin during the first three days after the operation, but not the slightest uneasiness. In this way the case progressed; the gum healed in a few days; the jaw united strongly, and in the time bones usually unite; and the wearing of the plate was discontinued within six weeks after the operation was performed. (Fig. 3 represents the manner of closing the jaw before the operation. Fig. 4, after the operation.)

FIG. 3.

FIG. 4.

The deformity of the jaw being now removed, the next thing to be done was to relieve the confined condition of the head, and the distortion of the face and neck resulting therefrom. This I determined to accomplish, if possible, after the manner of Prof. Mutter in similar cases; and I accordingly performed this operation on the 31st day of July, assisted by Dr. Frissell.

I began by dividing the skin immediately in front of the neck, about half an inch above the sternum—and then carried the incision back about three inches on each side. I then commenced a careful division of the strictures, which were so thickened in front as to extend to the trachea, and on the sides, as not only to involve the platysma-myodes, but a portion of the sterno-cleido-mastoideus muscles also. After dividing every thing that interfered with the raising of the head, and the closing of the mouth, so far as the incision was now made, it became evident that, to give free motion to the head, the incision on the neck must be extended back through the remaining cicatrix, which was at least two inches wide on one side, and about an inch and a half on the other; this was accordingly done, the whole presenting a wound upwards of nine inches in length, and nearly five in width. A thin piece of leather was now cut in the shape of the wound, but somewhat larger, and placing it upon the shoulder and arm, immediately over the deltoid muscle, a flap nearly ten inches in length, and five in breadth, having a neck or attachment two inches wide, was marked out and then dissected up as thick as the parts below would permit. This flap was now brought around, and secured in the wound on the neck by the twisted sutures; the sutures were placed about an inch and a half apart; between each of these sutures, one, two and sometimes three small stitches were inserted, depending entirely upon the number necessary to bring the edges neatly together. These stitches were of fine thread—had a very superficial hold, produced little or no irritation, and served to keep the parts in better apposition than any other means I could have devised. The wound on the shoulder was next drawn together about one-half of its entire extent; the remainder was covered with lint. One long narrow strip of adhesive plaster, applied around the neck to support the flap, and over this a cravat tied in the usual way, constituted all the dressing deemed advisable at this time.

The patient bore this tedious and very painful operation with great fortitude, and without uttering scarce a murmur. She was somewhat exhausted, but not from the loss of blood; there was no vessel divided of sufficient importance to require a ligature.

August 1st.—During the fore part of last night the patient was somewhat distressed—was very unmanageable—would talk incessantly, and occasionally sat up in bed. An anodyne was administered at 12 o'clock, after which she rested much better, and slept some. Complains of sickness of the stomach this morning—has vomited three or four times; flap very pale; pulse rather weak. Patient directed to refrain from taking all kinds of drink.

2d.—Patient complains of pain only in the shoulder; was much distressed the latter part of last night on account of a retention of urine. The catheter was employed, and about three pints of urine drawn off, after which she rested well. Pulse somewhat excited; flap better color.

3d.—The patient rested well last night—the use of the catheter still necessary. All efforts to keep the patient from talking and moving unavailing; color of the flap rather pale, save at the extreme point, and about two inches along the lower edge, which is assuming rather a dark blue color; pulse about the same as yesterday. Removed a pin from near the point of the flap, and enveloped the neck in cotton batting. Patient complains of hunger—chicken broth ordered.

4th.—Patient rested well; the use of the catheter still necessary; complains of slight head-ache; the color of the flap nearly natural, and even the point is assuming a healthy hue, and appears to be uniting; pulse almost natural.

5th.—Urinates without difficulty; bowels moved by injection; patient entirely free from pain; pulse natural.

6th.—Dressing removed; the flap is uniting by the first intention, along both sides, throughout its entire extent; the greater part of the pins and stitches removed.

7th.—The remainder of the pins and stitches removed; patient perfectly comfortable and cheerful.

10th.—Sat up all day by the window.

15th.—Walked out to take an airing.

During the whole progress of the cure there was not the slightest swelling or undue inflammation in the flap or about the neck. The patient was slightly hysterical the first few days, but never complained of any thing except pain on the shoulder, a slight head-ache of a few hours' duration, and the uneasiness occasioned by the retention of urine. The wound on the shoulder granulated rapidly, and skinned over in about six weeks after the operation. It was curious to observe that upon touching the flap after it had healed in the neck, the patient would always refer the sensation to the shoulder or arm from which the flap was taken.

The confinement of the head and distortion of the face occasioned by the strictures, being now removed, the next step was to relieve, as far as possible, a very great deformity of the under lip.

The under lip, from being dragged down and greatly stretched by the former projection of the under jaw, was rendered greatly too large—so much so that it pouted out an inch or more further than the upper lip. This, together with a turning out of the mucous membrane on the left side, which extended nearly down to the lower edge of the chin, making the lip too short on that side, was the nature of the deformity yet to be relieved.

To relieve this unseemly appearance of the lip, the inverted portion was cut out in a V shape, extending down to the flap in the neck, and sufficiently large to reduce the lip to the proper size. The edges were then brought together and secured after the manner of a single hare-lip. The wound healed in the most beautiful manner—the appearance of the lip was greatly improved, but still there remained a deep depression or notch in the edge, sufficiently large to keep exposed the tops of two or three teeth, besides preventing the coming together of the lips on that side.

I now determined to raise, if possible, this depressed portion of the lip, and for this purpose passed a bistoury through the lip, about two lines from the free edge, first on one side of the depression, and then on the other, and then carried the incisions downward, to meet at a point on the lower edge of the chin, as represented in Fig. 5.

FIG. 5.

The depressed portion of lip now lying between the two incisions was next dissected loose from the jaw, and then raised on a level with the remainder of the lip, and there retained by pins, after the manner of dressing a double hare-lip—the line of union forming the letter Y. (See Fig. 6.)

FIG. 6.

This operation was as successful as the others, and the original deformity being now removed, the young lady, though still carrying evidences of the burn, has the free use of her head, eye-lids, jaws and lips, and may mingle in society without particular note or remark. (Fig. 7 represents the patient before either of the operations were performed; Fig. 8, her appearance three weeks after the last operation.)

FIG. 7.

FIG. 8.

[The drawings of the first four cuts, accompanying this report, were procured through wax impressions of the mouth, and are therefore exact representations of the position of the teeth, and the manner in which the jaws closed together. The drawings of the last four cuts were taken from Daguerreotype likenesses. The Daguerreotype process, it is well known, *reverses the sides of the face*, and having neglected to direct the attention of the engraver to this fact, these cuts, though sufficiently faithful to give a very correct idea of the case in all other respects, represent the right for the left side of the face.]

WHEELING, *January 8th*, 1849.

## Figures and Tables

**Fig. 1. f1:**
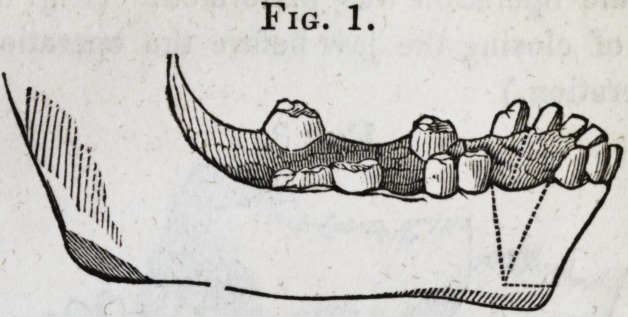


**Fig. 2. f2:**
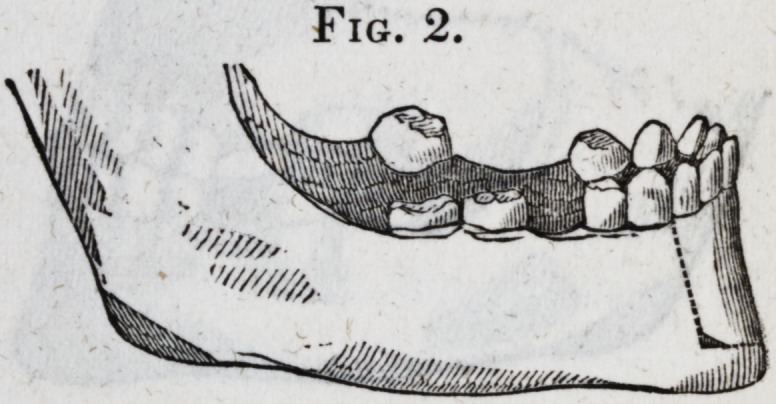


**Fig. 3. f3:**
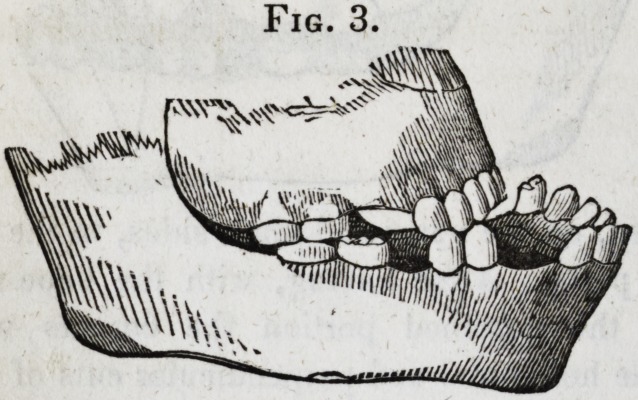


**Fig. 4. f4:**
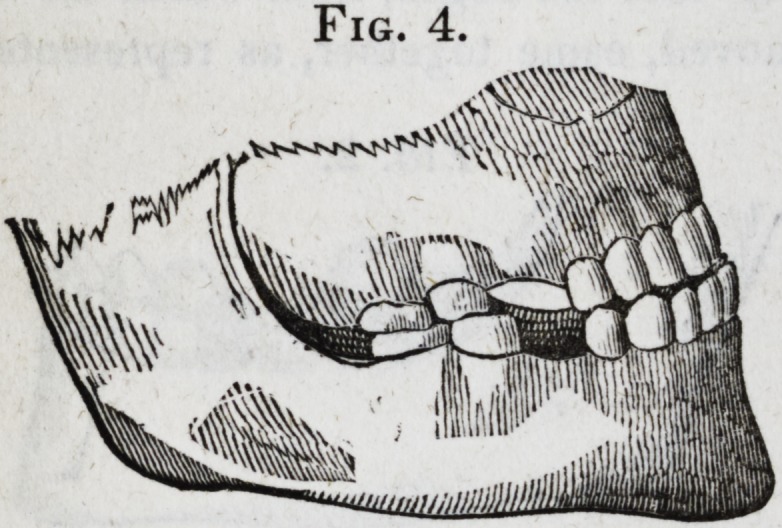


**Fig. 5. f5:**
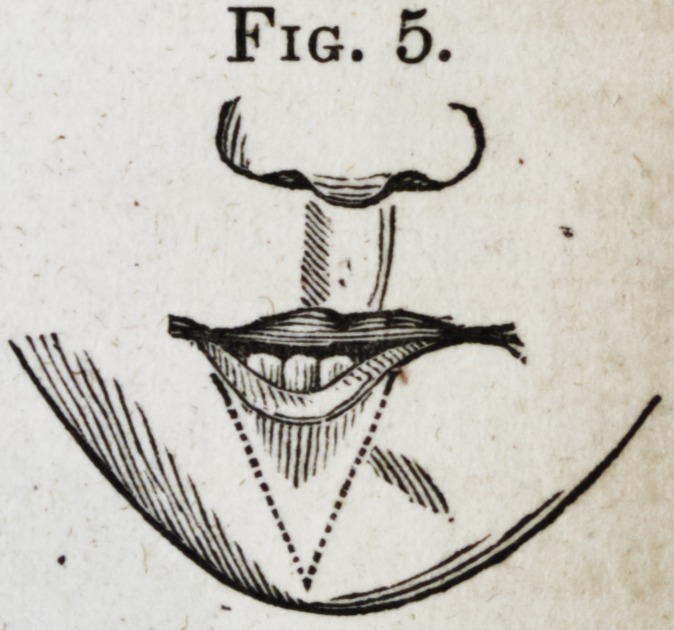


**Fig. 6. f6:**
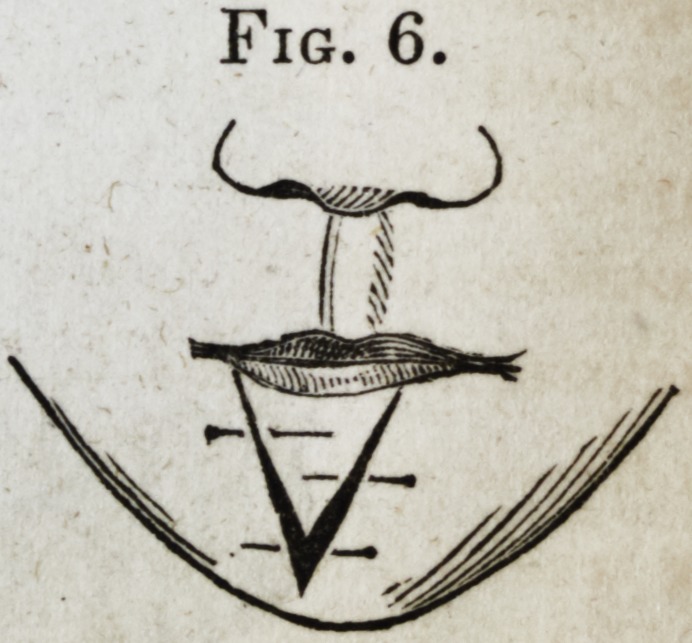


**Fig. 7. f7:**
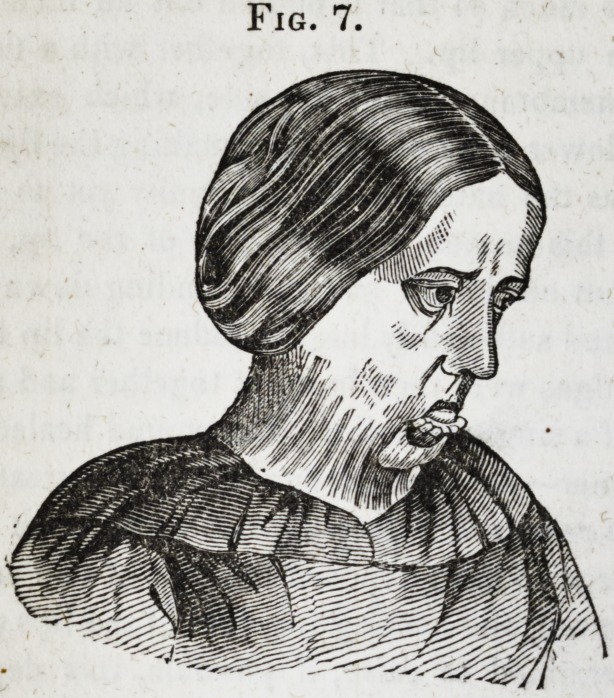


**Fig. 8. f8:**